# Reduced ELANE and SLPI expression compromises dental pulp cell activity

**DOI:** 10.1111/cpr.13132

**Published:** 2021-09-27

**Authors:** Kanokwan Sriwattanapong, Noppadol Sa‐Ard‐Iam, Lawan Boonprakong, Keskanya Subbalekha, Vorapat Trachoo, Narissara Suratannon, Thantrira Porntaveetus, Vorasuk Shotelersuk

**Affiliations:** ^1^ Genomics and Precision Dentistry Research Unit Department of Physiology Faculty of Dentistry Chulalongkorn University Bangkok Thailand; ^2^ Center of Excellence in Periodontal Disease and Dental Implant Immunology Research Center Faculty of Dentistry Chulalongkorn University Bangkok Thailand; ^3^ Oral Biology Research Center Faculty of Dentistry Chulalongkorn University Bangkok Thailand; ^4^ Department of Oral and Maxillofacial Surgery Faculty of Dentistry Chulalongkorn University Bangkok Thailand; ^5^ Pediatric Allergy & Clinical Immunology Research Unit Division of Allergy and Immunology Department of Pediatrics Faculty of Medicine King Chulalongkorn Memorial Hospital Chulalongkorn University Bangkok Thailand; ^6^ Center of Excellence for Medical Genomics Medical Genomics Cluster Department of Pediatrics Faculty of Medicine Chulalongkorn University Bangkok Thailand; ^7^ Excellence Center for Genomics and Precision Medicine King Chulalongkorn Memorial Hospital Bangkok Thailand

**Keywords:** cytokines, inflammation, neutropenia, neutrophil elastase, periodontitis, protease

## Abstract

**Background:**

Patients with *ELANE* variants and severe congenital neutropenia (SCN) commonly develop oral complications. Whether they are caused only by low neutrophil count or the combination of neutropenia and aberrant dental cells is unknown.

**Methods:**

Genetic variant was identified with exome sequencing. Dental pulp cells isolated from the SCN patient with an *ELANE* mutation were investigated for gene expression, enzyme activity, proliferation, colony formation, wound healing, apoptosis, ROS, attachment, spreading and response to lipopolysaccharide.

**Results:**

ELANE cells had diminished expression of *ELANE* and *SLPI* and reduced neutrophil elastase activity. Moreover, ELANE cells exhibited impaired proliferation, colony forming, migration, attachment and spreading; and significantly increased ROS formation and apoptosis, corresponding with increased *Cyclin D1* and *MMP2* levels. The intrinsic levels of *TGF*‐*β1* and *TNF*‐*α* were significantly increased; however, *IL*‐*6*, *IL*‐*8* and *NF*‐*kB1* were significantly decreased in ELANE cells compared with those in controls. After exposure to lipopolysaccharide, ELANE cells grew larger, progressed to more advanced cell spreading stages and showed significantly increased *SLPI*, *TNF*‐*α and NF*‐*kB1* and tremendously increased *IL*‐*6* and *IL*‐*8* expression, compared with controls.

**Conclusion:**

This study, for the first time, suggests that in addition to neutropenia, the aberrant levels and functions of *ELANE*, *SLPI* and their downstream molecules in pulp cells play an important role in oral complications in SCN patients. In addition, pulp cells with diminished neutrophil elastase and SLPI are highly responsive to inflammation.

## Graphical abstract

The dental pulp cells with diminished NE and SLPI, and *ELANE* variant exhibit impaired proliferation, colony forming, migration, attachment and spreading; significantly increased reactive oxygen species formation and apoptosis; and highly responsiveness to LPS‐induced inflammation. We show for the first time that ELANE, SLPI and their downstream molecules in dental pulp cells play an important role in cellular physiology and inflammatory response.

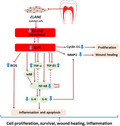

## 1 INTRODUCTION

Severe congenital neutropenia (SCN) is a heterogenous group of haematological disorders characterized by impaired promyelocytic proliferation and maturation with an absolute neutrophil count <500 cells/µL. Patients affected with SCN are prone to recurrent, often life‐threatening, infections that usually occur in the mucous membranes, skin and oral cavity.^1^ Neutrophils are the mainstay innate immune cells that are responsible for ensuring a healthy periodontal tissue.^2^ Common oral manifestations of SCN are gingivitis, periodontitis, ulcers and early tooth loss.^3^

More than 24 genes are associated with SCN; however, variants in *ELANE* account for >50% of SCN cases.^4^ *ELANE* encodes neutrophil elastase (NE), which exhibits antimicrobial effects. During infection, neutrophils release NE that cleaves the extracellular matrix protein elastin and modulates the expression of several cytokines, chemokines and growth factors.^5^ *ELANE* variants can cause NE misfolding, mistrafficking, and mislocalization, stress response pathway induction and promyelocyte apoptosis.^6^ Excess NE activity results in tissue damage.

A secretory leucocyte protease inhibitor (SLPI), a natural NE inhibitor, acts to counterbalance NE in a dose‐dependent manner.^7^ SLPI has multifunctional properties including antiprotease, antibacterial, antiviral and anti‐inflammatory functions.^8^, ^9^ SLPI is also involved in wound healing and cancer metastasis.^10^ Currently, the expression and pathogenic role of SLPI and ELANE in dental cells remain elusive.

The diseased cell phenotype is physiologically and molecularly different from that of healthy cells. They are good models to investigate molecular pathomechanisms and explore new and better biomarkers for early disease detection, including when signs and symptoms are barely discernable. We speculated that an imbalance in NE and SLPI might be involved in orodental inflammation and believed that the dental cells obtained from a patient with SCN and *ELANE* variant could be used to prove our hypothesis. Here, we identified that the diminished *ELANE* and *SLPI* expression, as well as NE activity regulate the behaviour, survival and inflammatory responses of dental pulp cells. After exposure to liposaccharide, the patient's pulp cells exhibited significantly higher inflammatory cytokine expression. Our findings suggest that ELANE and SLPI are important regulators in the inflammatory response of dental pulp cells.

## 2 MATERIALS AND METHODS

### 2.1 Subject

This study was approved by the Institutional Review Board, Faculty of Medicine, Chulalongkorn University (IRB 316/63) and in accordance with the 1964 Helsinki Declaration and its later amendments. Written informed consent was obtained from the patient or a legal guardian. Genotype and dental phenotypes analyses were performed as previously described.^11^, ^12^, ^13^

### 2.2 Mutation analysis

Genomic DNA was extracted from peripheral blood leucocytes of the patients using Puregene Blood Kit (Qiagen) and sent for exome sequencing using lllumina Hiseq 2000 Sequencer (Macrogen). The sequences were aligned to the University of California Santa Cruz (UCSC) hg19 using Burrows‐Wheeler Aligner (http://bio‐bwa.sourceforge.net/). Downstream process was performed by SAMtools (samtools.sourceforge.net/) and annotated against dbSNP and 1000 Genomes. The variants were filtered using the following criteria: (a) located in exons or flanking introns of the genes related to neutropenia (HPO HP:0001875); (b) minor allele frequency <1% in 1000 Genomes Project, The Genome Aggregation Database (gnomAD: gnomad.broadinstitute.org); (c) not presented in the in‐house database of 2166 unrelated Thai exomes; (d) not synonymous exonic variants; and (e) predicted to be pathogenic by prediction software. The identified variant was verified by PCR‐Sanger sequencing.

### 2.3 Characterization of a patient's teeth

Clinical and radiographic examination were performed. The patient's deciduous upper central incisor was extracted due to tooth mobility and scanned using specimen micro‐computed tomography and compared with three incisors from age‐matched healthy individuals. After the pulp tissues were explanted, the tooth samples were scanned with Specimen Micro‐CT35 (SCANCO Medical). Ten spots in the enamel and ten spots in the dentin areas were selected to quantify their mineral densities.

### 2.4 Cellular and molecular characteristics of ELANE dental pulp cells

Dental pulp cells were isolated from the patient's deciduous upper central incisors (ELANE cells). They were investigated in comparison with three lines of dental pulp cells from the deciduous incisors of age‐matched healthy individuals. Briefly, the teeth were rinsed with PBS. The pulp tissues were removed and cut into 1 × 1 mm pieces and placed in a 35‐mm culture dish (Corning). The explants were maintained in growth medium composed of Dulbecco's Modified Eagle Medium (DMEM) containing 10% foetal bovine serum (Gibco), 1% L‐glutamine (Gibco) and 1% penicillin and streptomycin (Gibco), incubated in a humidified environment at 37℃ and 5% CO_2_. Cells from passages 4–6 were used in the experiments. All experiments were performed at least three times.

### 2.5 Mesenchymal markers

The isolated cells were characterized using flow cytometry. The expression of the surface markers CD44 (Cat No. AM310‐10 M, BioGenex), CD45 (Cat No. AM111‐10 M, BioGenex), CD90 (Cat No. 21270906, ImmunoTools), CD105 (Cat no. 21271054, ImmunoTools) and CD73 (Cat no. 21270733, ImmunoTools) was investigated.

### 2.6 Immunofluorescence microscopy

Cells were cultured on a Chamber Slide^TM^ at density 5 × 10^3^ cells/well, with/without 0.1 µg/mL lipopolysaccharide (LPS) for 24 h. The cells were washed and fixed in 4% ice‐cold paraformaldehyde at room temperature for 10 min, then rinsed three times for 5 min in PBST consisting of 10 mM phosphate‐buffered saline (PBS) and 0.1% tritonX100 (Sigma‐Aldrich). Non‐specific binding sites were blocked by incubating the cells in 1% bovine serum albumin in PBS. Neutrophil elastase (NE) primary antibody (Cat No.#MAB91671, R&D system; 1:200) was diluted in blocking buffer and incubated overnight at 4℃. After washing three times, the cells were incubated with Alexa 488 goat anti‐mouse IgG antibodies (Cat No.#405319, BioLegand; 1:1000), counterstained with rhodamine‐phalloidin (Invitrogen, Carlsbad; 1:1000) diluted in blocking buffer for 1 h at room temperature in the dark and mounted with VECTASHIELD^®^ Antifade Mounting Medium with DAPI (Cat No. H‐1200, Vector Laboratories). Immunofluorescence images were taken using a fluorescence microscope (ZEISS).

### 2.7 Western blot analysis

Confluent monolayer cells were harvested, washed with ice‐cold phosphate‐buffered saline (PBS) and lysed in radioimmunoprecipitation buffer (RIPA) (Thermo Fisher Scientific) containing a Halt^TM^ protease inhibitor cocktail (Thermo Fisher Scientific). Protein concentration was determined using Pierce^TM^ BCA Protein Assay Kit (Thermo Fisher Scientific). A total 30 µg protein were separated by 12.5% sodium dodecyl sulphate‐polyacrylamide gel electrophoresis and transferred to a polyvinylidene difluoride membrane (PVDF) (Bio‐Rad). The PVDF membranes were blocked with 5% BSA (Merck Millipore, Germany) in TBST buffer (10 mM Tris‐HCl pH 8.0, 150 mM NaCl and 0.1% Tween‐20) for 1 h at room temperature. Primary antibodies were blotted overnight at 4℃. Three membranes were blotted with mouse anti‐human NE monoclonal antibody (R&D systems; 1:2500), followed by mouse anti‐GAPDH monoclonal antibody (Abcam; 1:3000). The other three membranes were blotted with goat anti‐human SLPI polyclonal antibody (R&D systems; 1:1000), followed by mouse anti‐GAPDH monoclonal antibody. The rabbit anti‐goat or goat anti‐mouse HRP‐conjugated secondary antibodies were incubated (R&D systems; 1:2500) for 2 h at room temperature. The membranes were treated with SuperSignal^TM^ West Femto Maximum Sensitivity Substrate (Thermo Fisher Scientific) and analysed by the Amersham^TM^ Imager 680 (GE Healthcare). The intensity of the bands was quantified using ImageJ software.

### 2.8 Neutrophil elastase (NE) activity

NE activity was measured using a fluorometric neutrophil elastase activity assay kit (Cat# ab204730, Abcam) following modified manufacturer's instructions. A single‐cell suspension of ELANE cells and controls were seeded with/without 0.1 µg/mL of LPS in 96‐well plates using non‐phenol red DMEM with 10% FBS at a density of 2 × 10^4^ cells/well and cultured at 37℃ in 5% CO_2_ for 24 h. After 24 h, neutrophil elastase substrate was added into each well. The activity was measured using fluorescent microplate readers at Ex/Em = 380/500 nm in a kinetic mode, every 2 min at 37℃ for 10–20 min. Cells with no substrate added were used as a background control. The amount of NE was calculated: ΔRFU_380/500nm_ = (RFU_2_‐RFU_BG_) ‐(RFU_1_‐RFU_BG_). RFU_1_, the sample reading at time T_1_; RFU_2_, the sample reading at time T_2_; RFU_BG_, the background control sample.

### 2.9 Real‐time polymerase chain reaction (Real‐time PCR)

Total cellular RNA was isolated by RiboEx total RNA isolation solution (GeneAll). The concentration of the isolated RNA was measured using Thermo Scientific Nanodrop one (Thermo Fisher scientific). iScript Reverse Transcription (Bio‐rad) was used for converting RNA into cDNA. Real‐time PCR was performed with SYBR green detection system (FastStart Essential DNA Green Master, Roche Diagnostic) and MiniOpticon real‐time PCR system (Bio‐Rad, Hercules). Primer sequences are shown in Table S1.

### 2.10 Proliferation assay

Cells were seeded at 5 × 10^3^ cell per well in 48‐well plates. After 3, 7 and 10 days, 3‐(4,5‐dimethylthiazol‐2‐yl)‐2,5‐diphenyltetrazolium bromide (MTT) (Merck, Darmstadt, Germany) was added to the cells at a final concentration of 0.5 mg/mL. Cells were further incubated for 1 h at 37ºC. After removing the supernatant, the MTT crystals were dissolved using a mixture of glycine and DMSO (1:10). The formazan colour was determined spectrophotometrically at 570 nm.

### 2.11 Colony‐forming assay

Single‐cell suspensions (passages 4–6) were seeded in six‐well plates at a density of 300 cells per well and cultured in DMEM with 10% FBS at 37℃ in 5% CO_2_. After 14 days, the cells were fixed in 100% methanol for 10 min and stained with 0.1% crystal violet. Colonies containing more than 50 cells were counted.

### 2.12 Wound healing assay

Cells were cultured in 12‐well plates at a density of 1 × 10^4^ cells per well in triplicate until a confluent monolayer was formed. A 200‐µL pipette tip was used to make a scratch approximately 100 µm wide. The cells were then washed with phosphate‐buffered saline (PBS) and cultured in DMEM medium with 1% FBS at 37℃ for 48 h. Micro‐photographs were taken at 0, 24 and 48 h after scratching using a Zeiss microscope equipped with a digital camera. The scratch‐wound closure percentage was determined using ImageJ software. The wound healing rate (%) = ((the area of primary wounds–the area of healing)/the area of primary wounds)) x 100.

### 2.13 Cell apoptosis

Cell apoptotic rate was detected using a FITC Annexin V apoptosis detection kit with propidium iodide (PI) (Cat#640914, BioLegend) following the manufacturer's instructions. Briefly, confluent monolayer cells were harvested and washed with PBS. Then, the cells were re‐suspended in 100 µL binding buffer containing 5 µL Annexin V‐FITC and 10 µL PI, incubated for another 15 min at room temperature in the dark, and 300 µL binding buffer was added. Flow cytometric analysis was performed using FACSCalibur flow cytometer (Becton Dickinson).

### 2.14 Reactive Oxygen Species (ROS) formation

ROS formation was detected by the oxidation of 2’,7’‐dichlorogluorescin diacetate (DCFDA) (Sigma‐Aldrich, St. Louis) into a highly fluorescent compound, 2’,7’‐dichlorofluorescien (DCF). Cells were plated at a density of 1 × 10^4^ cells/well in 12‐well plates and incubated until ~90% confluence. Before removing the media, the cells were incubated with 10 μM DCFDA for 30 min at 37℃. The cells were then collected, washed with PBS and analysed by FACSCalibur flow cytometer (Becton Dickinson).

### 2.15 Cell attachment and spreading assay

Cells were cultured on glass coverslips for 30 min, 2 h, 6 h and 24 h with/without 0.1 μg/mL of LPS. After each incubation time point, the cells were washed with PBS and fixed with 3% glutaraldehyde (Sigma‐Aldrish) for 30 min at room temperature. Cells were dehydrated in graded series of ethanol, hexamethyldisilazane (HMDS), sputter‐coated with gold and analysed with a scanning electron microscope (Quanta 250, FEI). The cell spreading category criteria were previously reported.^14^

### 2.16 Statistical analyses

The experiments were performed at least three times. Two‐group comparison was determined using Mann‐Whitney U test (Prism5, GraphPad Software). The *p* value less than 0.05 was considered to be statistically significant (*p* < 0.05(*), *p* < 0.005(**), *p* < 0.0005(***)). The Mann‐Whitney U test was chosen because it is distribution‐free and proper for experiments with small sample sizes.

## 3 RESULTS

### 3.1 Clinical investigations

**FIGURE 1:**
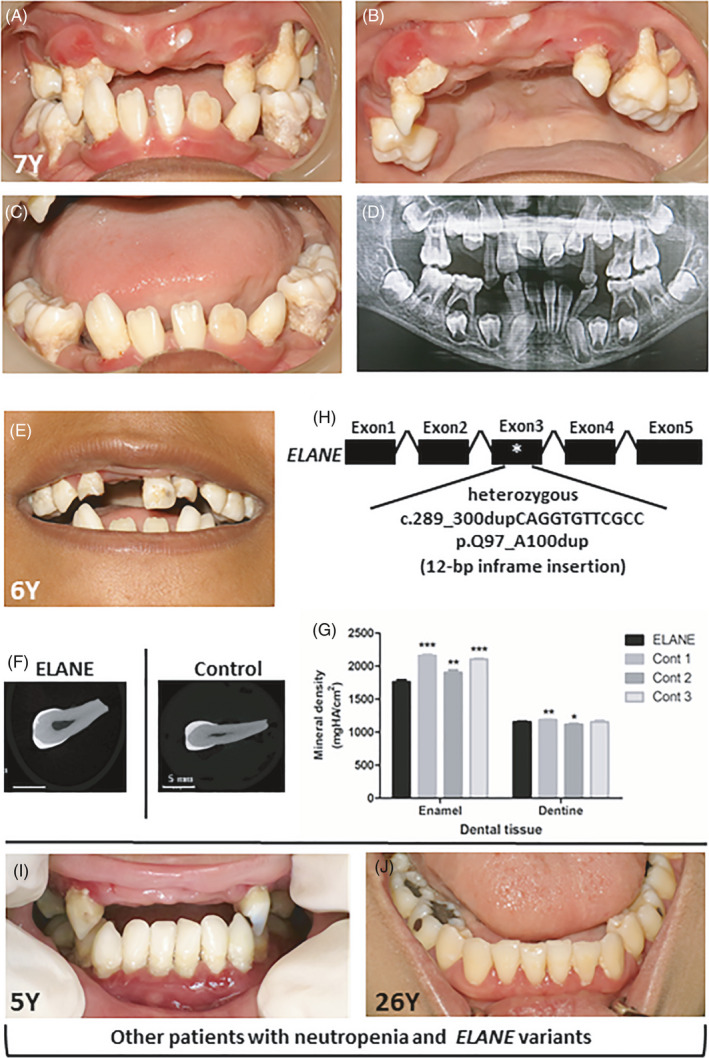
Oral phenotype and genetic mutation in a SCN patient. (A‐C) Oral photographs of the patient at 7 years old demonstrated severe periodontitis, dental infection, ulcers, dental caries, tooth mobility and premature tooth loss. (D) Panoramic radiograph of the patient demonstrated alveolar bone destruction, malocclusion, tooth extrusion and premature loss of deciduous teeth. (E) The photograph of the patient at 6 years old illustrated a deciduous upper left central incisor with enamel hypomineralization. (F) Micro‐computed tomography (Micro‐CT) images of the deciduous upper left central incisor of the patient and control. (G) Micro‐CT analysis revealed that the mineral density of the patient's tooth enamel was significantly less than that of controls. (H) The patient was identified with the heterozygous 12‐bp inframe insertion, c.289_300dupCAGGTGTTCGCC; p.Q97_A100dup, in *ELANE*. (I, J) The other two unrelated patients who had neutropenia and *ELANE* variants showed tooth infection, hypomineralized enamel and gingival inflammation

The patient was a 7‐year‐old girl. She was diagnosed with severe congenital neutropenia (SCN) at 10 months old due to persistent neutropenia and decreased myeloid series with arrested myelocyte maturation in the bone marrow. The patient's medical condition and genotype were previously reported.^13^ Briefly, the patient had regularly received granulocyte‐colony stimulating agents (G‐CSF), but still experienced recurrent infections, including gastroenteritis, perineal abscess, otitis media, mastoiditis, maxillary and ethmoidal sinusitis, and temporal scalp abscess. Oral examination at 7 years of age revealed generalized severe periodontitis (Stage IV, Grade C), oral infection, ulcers, malocclusion and several mobile teeth. The teeth demonstrated enamel hypomineralization, dental caries and root exposure. The erupting permanent lower lateral incisor also had enamel defects that were likely to result from the infected deciduous incisor (Figure 1A‐C). In addition to the proband, we examined other patients with *ELANE* mutations and neutrophil defects. They all had orodental problems, including tooth infection, gingival inflammation, periodontal disease, hypomineralized enamel and dental caries (Figure 1I, J). Panoramic radiographs revealed periapical infection, furcation involvement and advanced alveolar bone loss (Figure 1D). The deciduous upper left central incisor was extracted due to mobility at 6 years of age (Figure 1E). Microscopic images demonstrated hypomineralized spots on the enamel (Figure 1F). Micro‐CT revealed significantly reduced mineral density in the patient's enamel compared with that of controls (Figure 1G). She was identified with a heterozygous 12‐bp inframe insertion, c.289_300dupCAGGTGTTCGCC; p.Q97_A100dup, in exon 3 of the *ELANE* gene (OMIM *130130) (Figure 1H). This mutation was reported in our previous study.^13^ The diagnosis of autosomal dominant SCN 1 (OMIM # 202700) was confirmed.

### 3.2 Severely downregulated ELANE and SLPI expression, and NE activity

**FIGURE 2:**
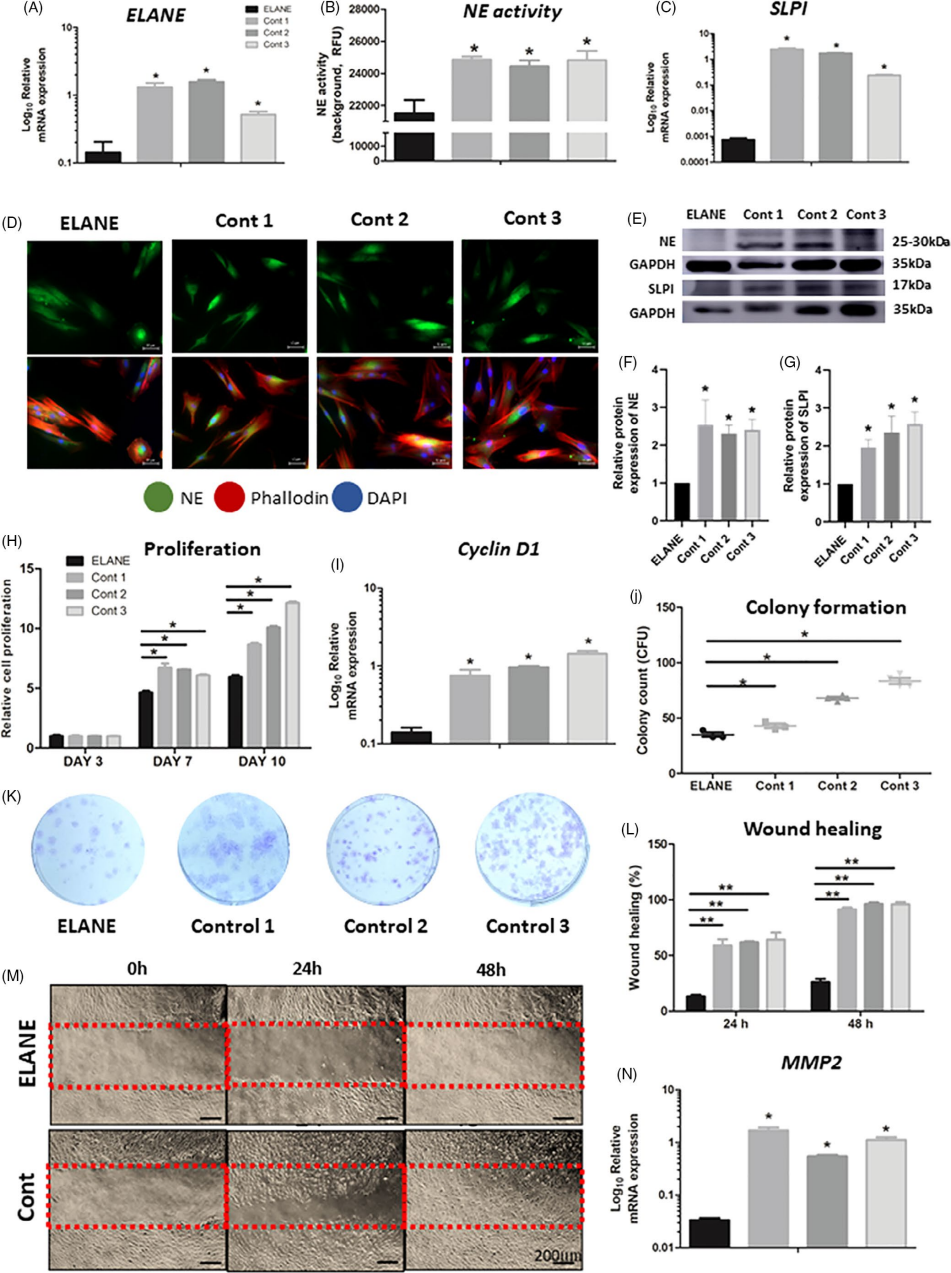
Gene and protein expression, enzyme activity, proliferation, colony formation and wound healing. (A‐C) The mRNA expression of *ELANE* and *SLPI*, and neutrophil elastase (NE) activity of ELANE cells were significantly less than those of control. (D) Immunofluorescence detected NE staining in ELANE cells compared with controls. NE/ neutrophil elastase (green); Phalloidin (red); DAPI (blue). (E‐G) The NE and SLPI protein expression was significantly reduced in ELANE cells compared with that in controls. The GAPDH bands denote the controls for the NE or SLPI bands located above. (H) MTT assay showed that ELANE cells are significantly less proliferative than controls at Day 7 and Day 10. (I) The *Cyclin D1* mRNA expression was significantly reduced in ELANE cells. (J, K) ELANE cells presented significantly less colony formation than controls. (L‐N) ELANE cells had delayed migration and wound healing and reduced MMP2 expression compared with controls. A significant difference between ELANE cells and controls: *p < 0.05; **p < 0.005; ***p < 0.0005. Cont, Control

ELANE cells and controls expressed mesenchymal stem cell markers comprising CD45, CD73, CD90 and CD105, but not CD45, indicating that these cells were mesenchymal cells (Figure S1). We investigated whether *ELANE* mutation affected *ELANE* expression in the dental pulp cells of the SCN patient and observed that ELANE cells had a significantly diminished *ELANE* expression compared to controls (Figure 2A). Correspondingly, the fluorometric neutrophil elastase activity assay indicated that ELANE cells had significantly decreased NE activity compared with controls (Figure 2B). Western blots showed that the NE protein level was significantly reduced in ELANE cells compared with that in controls (Figure 2E, F, Figure S3, Table S2), although the immunofluorescence showed no significant difference in NE expression between ELANE cells and controls (Figure 2D). Furthermore, ELANE cells exhibited significantly downregulated mRNA and protein expression of SLPI, the natural inhibitor of neutrophil elastase (Figure 2C, E, G, Figure S3, Table S2).

### 3.3 Impaired proliferation, colony formation and migration

SLPI is involved in multiple aspects of cell behaviours including proliferation, migration and wound healing. Because *ELANE* and *SLPI* were significantly downregulated in ELANE cells, we investigated whether ELANE and SLPI were essential for dental pulp cells survival. An MTT assay demonstrated that ELANE cells had proliferation comparable to controls at Day 3. Significantly compromised proliferation was observed in ELANE cells compared with controls at Day 7, and this significant difference was more pronounced at Day 10 (Figure 2H). Accordingly, the mRNA expression of *Cyclin D1*, a gene involved in cell proliferation, was significantly reduced in ELANE cells compared with controls (Figure 2I). In addition, the colony‐forming unit (CFU) assay illustrated that the ELANE cell CFUs were significantly fewer compared with controls (Figure 2J, K). We next evaluated cell migration and wound healing using a scratch wound assay. The assay demonstrated a significantly reduced wound healing percentage in ELANE cells compared with controls at 24 h and 48 h (Figure 2L, M and Figure S4). Cell migration relies on the synthesis and secretion of proteolytically active matrix‐metalloproteinases (MMPs). Thus, we examined the mRNA expression of *MMP*2, which participates in cell migration under various physiological/pathological conditions and observed a significantly reduced *MMP2* level in ELANE cells compared with controls (Figure 2N).

### 3.4 Induced apoptosis and reactive oxygen species (ROS) formation

**FIGURE 3:**
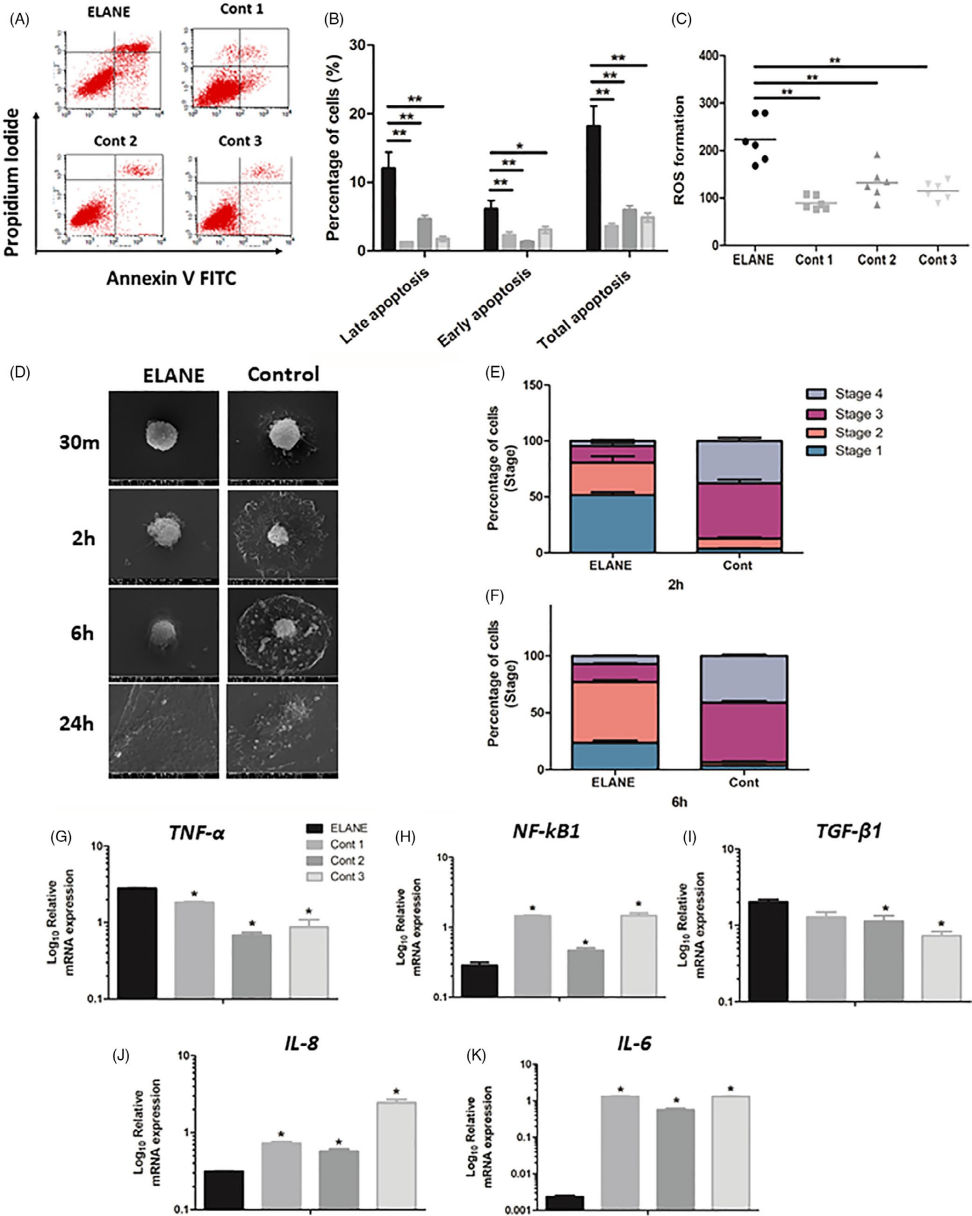
Apoptosis, attachment, migration and inflammatory genes expression. (A‐C) ELANE cells showed significant increases in cell apoptosis and reactive oxygen species (ROS) formation. (D‐F) ELANE cells had delayed cell stages. (G‐K) The mRNA expression of *TNF*‐*α* and *TGF*‐*ß1* was significantly increased, while *NF*‐*kB1*, *IL*‐*6* and *IL*‐*8* were significantly decreased in ELANE cells compared with controls. A significant difference between ELANE cells and controls: *p < 0.05; **p < 0.005; ***p < 0.0005. Cont, Control

We speculated that the attenuated proliferation, colony forming and migration in ELANE cells was associated with increased apoptosis and ROS formation. We observed that ELANE cells had a higher number of apoptotic (annexin V‐positive) and a higher number of dead cells (annexin/propidium iodide‐positive) than controls (Figure 3A). ELANE cells exhibited significantly higher percentages of cells in early, late and total apoptosis compared with controls (Figure 3B). ROS formation was also significantly increased in ELANE cells compared with controls (Figure 3C and Figure S2).

### 3.5 Reduced cell attachment and spreading

Cell attachment and spreading are important for cell communication in tissue development and maintenance. Altered cell attachment can lead to tissue destruction as reported in many diseases and cancers.^15^ Thus, we analysed whether the reduced ELANE cell proliferation was associated with delayed cell attachment and spreading. In vitro cell attachment and spreading were evaluated using scanning electron microscopy. At 30 min, ELANE cells were round with filopodia and/or lamellopodia that were shorter than those of controls. At 2 h and 6 h, control cells formed extended circumferential lamellipodia, while ELANE cells exhibited a domelike morphology and minimally extended lamellipodia. At 24 h, the ELANE and control cells were flattened, indicating complete cell spreading (Figure 3D and Figure S5).

Next, we compared the percentage of ELANE and control cells in each cell attachment stage and observed that the majority of ELANE cells at 2 h were at stage 1, while the majority of controls were at stage 3 (Figure 3E). At 6 h, most ELANE cells were at stage 2, while most controls had entered stage 3 and 4 (Figure 3F).

### 3.6 Altered inflammatory profiles

Defects in neutrophils, the central immune regulators and their antimicrobial activities can impact the inflammatory profiles of other cells and tissue homeostasis. We observed that the ELANE cells exhibited significantly higher *TNF*‐*α* and *TGF*‐*ß1*, but lower *NF*‐*kB1*, *IL*‐*6* and *IL*‐*8* expression levels compared with controls (Figure 3G‐K).

### 3.7 Upregulated inflammatory responses to LPS exposure

**FIGURE 4:**
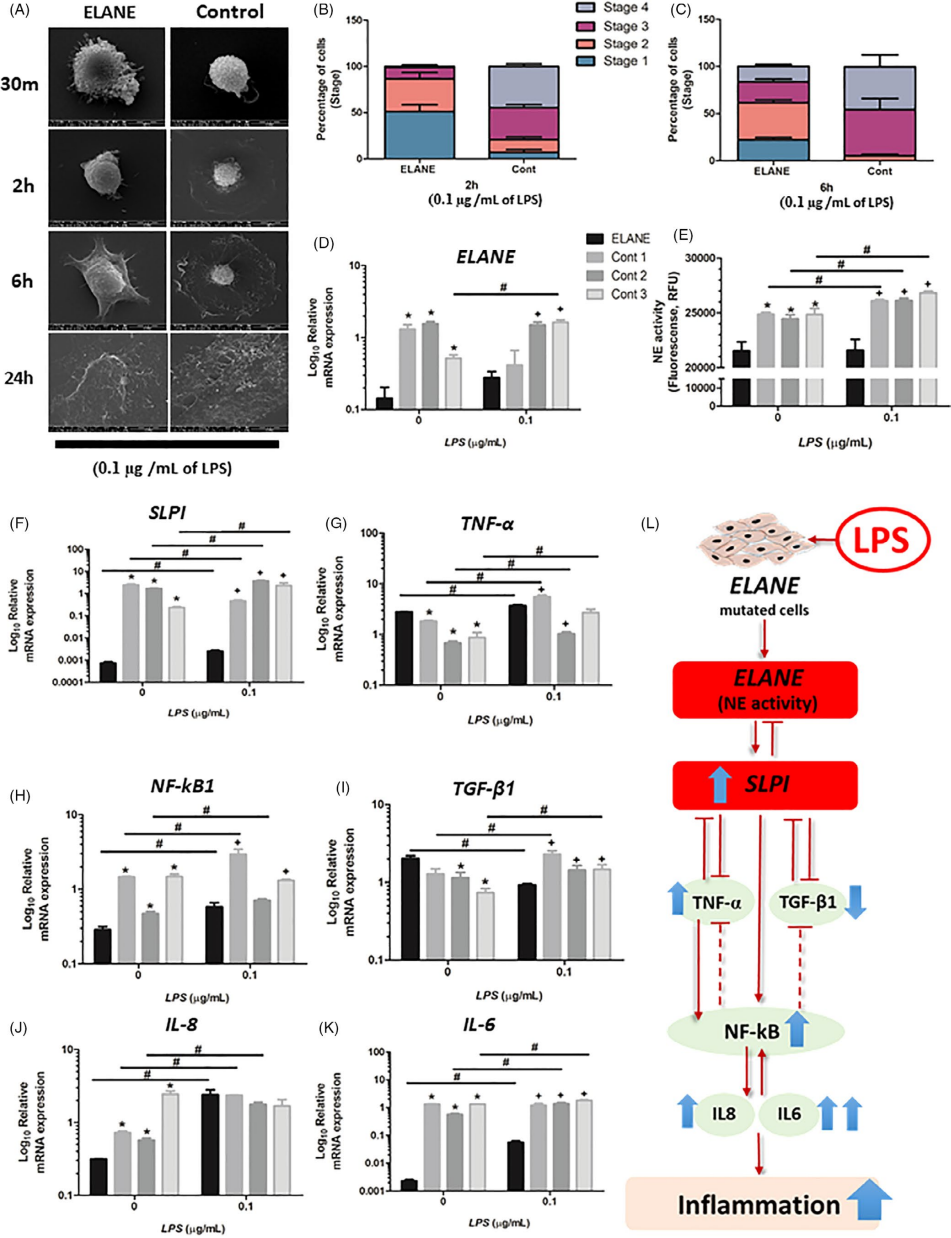
LPS response. (A) LPS‐treated ELANE cells grew bigger than controls. (B, C) Treated ELANE cells and treated controls progressed faster than untreated cells. (D, E) No significant difference in *ELANE* expression and NE activity between treated and untreated ELANE cells. Treated cells showed significantly increased NE activities compared with untreated cells. (E‐K) The mRNA expression of *SLPI*, *TNF*‐*α*, *NF*‐*kB1*, *IL*‐*6* and *IL*‐*8* was significantly upregulated in LPS‐treated ELANE cells. (I) *TGF*‐*ß1* was significantly decreased in treated ELANE cells compared with untreated, whereas an increased *TGF*‐*ß1* level was observed in treated controls. (L) Schematic diagram showing how LPS affects gene expression in ELANE cells. *A significant difference between nontreated ELANE cells and nontreated controls; +A significant difference between LPS‐treated ELANE cells and LPS‐treated controls; #A significant difference between LPS‐treated cells and untreated cells. *, +, #*p* < 0.05. Cont, Control

To further investigate how ELANE cells react to inflammation; the cells were treated with lipopolysaccharide (LPS) which is the most potent bacterial cell wall‐derived inflammatory toxin. When treated with LPS, ELANE cells grew larger compared with controls and untreated cells (Figures  4A, 3D, and Figure S6). Cell staging showed that both LPS‐treated ELANE cells and controls progressed faster at 2 h and 6 h compared with untreated cells (Figure  4B, C and Table S3). LPS‐treated ELANE cells showed a nonsignificant increase in *ELANE* expression and NE activity compared with untreated cells, while NE activities in the treated controls significantly increased (Figure 4D, E). The *SLPI* levels in treated ELANE cells and controls were significantly upregulated compared with their corresponding untreated cells; however, the level in treated ELANE cells was significantly lower than those in treated controls (Figure 4F).

Regarding inflammatory gene marker expression, the LPS‐treated ELANE and control cells exhibited significantly higher *TNF*‐*α* expression compared with untreated cells (Figure 4G). The *NF*‐*kB1* levels after LPS treatment were significantly increased in all cells except one control (Figure4H). Conversely, LPS‐treated ELANE cells exhibited lower *TGF*‐*ß1* expression (0.46‐fold change), while controls had a higher expression than untreated cells (1.67‐fold change) (Figure 4I and Table S4). Untreated ELANE cells exhibited significantly much lower *IL*‐*6* and *IL*‐*8* expression compared with untreated controls. However, after LPS exposure, IL‐6 and IL‐8 levels were dramatically upregulated (24.07‐fold and 7.67‐fold increases respectively) in treated ELANE cells, compared with controls (1.57‐fold and 2.34‐fold) (Figure 4J, K and Table S4).

## 4 DISCUSSION

Neutrophils are the major immune cells that play a critical role in the acute inflammatory response and host defences against bacterial infections. The consequence of neutropenia is increased infection susceptibility. Phenotypically, the prime features associated with SCN are dental and periodontal inflammation. Other orodental findings are recurrent dental infection, mouth ulcers, hypomineralized enamel and dental caries. Genotypically, the patient was identified with a *de novo* heterozygous 12‐bp inframe insertion in the *ELANE* gene. This mutation is located in the peptidase S1 domain. Our genotype‐phenotype correlation found that the SCN patients who harboured *ELANE* variants had more severe periodontal diseases compared with the cyclic neutropenia patients with *ELANE* variants or the SCN patients with variants in other genes, such as *HAX1*.^16^ Correspondingly, our SCN patient had early‐onset and rapidly progressive periodontitis resulting in severe tooth mobility and premature tooth loss.

Neutrophils defend against microbes by several mechanisms; phagocytosis, degranulation and releasing neutrophil serine proteases. To kill ingested microorganisms, neutrophils use oxygen‐dependent and oxygen‐independent mechanisms. In the oxygen‐dependent bactericidal activity, pathogen phagocytosis leads to superoxide production by NADPH oxidase and ROS formation, while their oxygen‐independent activity involves the release of antimicrobial peptides, including neutrophil elastase (NE), proteinase 3 and cathepsins. Reduced NE activity was associated with recurrent infections in SCN patients.^17^, ^18^ However, the uncontrolled production of ROS and enzymes from neutrophils can damage adjacent host cells and tissues.^19^ To counteract this, SLPI, a natural inhibitor of NE, functions to counteract the protease activity of neutrophils in a reciprocal manner, where NE stimulates *SLPI* expression. Treating myeloid cells with exogenous NE resulted in upregulated SLPI expression, whereas NE inhibition downregulated SLPI expression.^17^ NE and SLPI expression are severely downregulated in SCN patients.^17^

To date, the pathophysiology by which NE and SLPI are related to dental infection is unclear. Here, we show a possible pathomechanism between ELANE mutation, diminished NE and SLPI expression, and dental inflammation in ELANE dental pulp cells. We demonstrate that *ELANE* and *SLPI* levels are severely downregulated in the dental pulp cells of a SCN patient with an *ELANE* mutation compared with controls. The ELANE cells presented cellular pathophysiology comprising compromised cell proliferation, colony formation, migration, wound healing, apoptosis and anti‐inflammatory capacities.

NE and SLPI are involved in many cellular pathways, including cell proliferation, colony formation, cell migration, ROS formation and cell apoptosis.^6^, ^20^ Cell attachment and spreading are important for cell and tissue morphogenesis and inflammatory response.^21^ Extracellular matrix glycoprotein interactions promote cell adhesion and cytoskeletal reorganization, which also regulate cell proliferation, migration and differentiation. Cell death triggers adhesion protein cleavage that can interrupt cell behaviour.^22^ Primary granulocytes expressing mutant NE protein exhibited a significant increase in apoptosis due to an unfolded protein response, ER stress and intracellular ROS generation.^23^ SLPI inhibits neutrophil apoptosis by reducing p38 MAPK activation and pro‐apoptosis protein (BAX) levels, and preventing ROS formation.^24^, ^25^ SLPI reduces intracellular ROS production and provides cardio‐vasculoprotective effects against ischaemia/reperfusion injury.^26^ Downregulated SLPI expression results in cell‐cycle arrest and elevated apoptosis.^17^ In the present study, the downregulated *SLPI* expression observed in ELANE cells could lead to increased intracellular ROS formation and cell apoptosis. Moreover, *SLPI* specifically escalates *Cyclin D1* gene expression by activating its transcription promoter, leading to cell proliferation.^27^ Transfected SLPI‐siRNA in gastric cancer cell lines resulted in significantly reduced *MMP2* and *MMP9* expression, as well as cell migration and invasion rates.^24^, ^28^ SLPI‐null mice have altered inflammatory responses, for example enhanced *TGF*‐*β* activation, delayed wound healing and an increased and prolonged inflammatory response.^29^ The consequence of *SLPI* deficiency are impaired wound healing and increased inflammation and elastase activity.^10^ Consistent with this evidence, in addition to NE and SLPI downregulation, we observed that ELANE cells had impaired proliferation, colony formation, migration, attachment and spreading. Moreover, apoptosis and ROS generation were increased, and *Cyclin D2* and *MMP2* expression was decreased. These altered cell behaviours and extracellular matrix interaction by ELANE cells can disrupt normal physiology and implicate it in the pathogenesis of dental diseases. It is therefore possible that, in SCN patients, myeloid progenitors that undergo apoptosis and dental pulp cells might have a similar pathophysiology.

Signalling cross‐talk between TGF‐β, NF‐κB and other signalling pathways play critical roles in tissue homeostasis by regulating cell proliferation, survival and differentiation. Regarding TGF‐β signalling, SLPI expression has a reverse correlation with *TGF*‐*β* expression.^30^ Unregulated TGF‐β expression is associated with downregulated SLPI expression and consequently inhibited NE activity.^31^ *TGF*‐*β1* induces apoptosis through *smad2*/*3*/*4*.^32^ In TNF signalling, *TNF*‐*α* binds to the TNF receptor 2 to activate NF‐kB1 and promote cell survival and binds to TNF receptor 1 to activate caspase‐3 in a death signal, leading to DNA degradation and apoptosis. Thus, dysregulated *TGF*‐*β1* and *TNF*‐*α* expression is linked with apoptosis induction.^9^, ^33^ SLPI also inhibits the TNF‐α‐induced caspase‐3 activation associated with apoptosis in monoblast cell lines and peripheral blood monocytes.^9^ These findings indicate that the reduced *SLPI* expression in ELANE cells could lead to apoptosis through upregulated *TGF*‐*β1* and *TNF*‐α expression together with increased ROS production.

**FIGURE 5:**
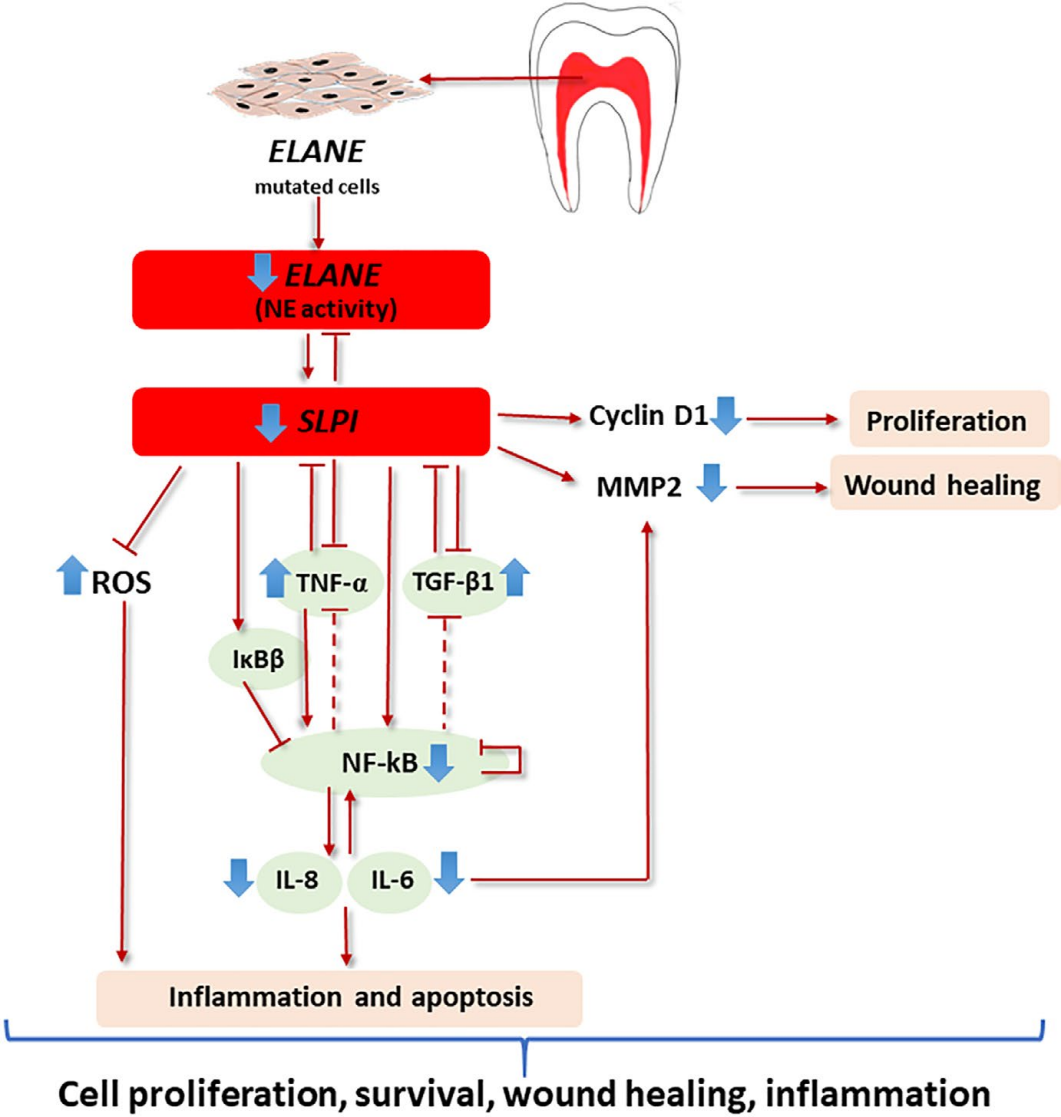
Schematic diagram showing how reduced ELANE and SLPI expression affects the cellular and molecular features of dental pulp cells

NF‐κB serves as a central inflammatory mediator, and its dysregulation results in various inflammatory diseases. SLPI modulates inflammatory cytokines, such as TNF‐α, TGF‐β1, NF‐kB1, IL‐6 and IL‐8, which are involved in NF‐κB and TGF‐β signalling.^34^ In the NF‐κB pathway, SLPI has an anti‐inflammatory effect by downregulating *TNF*‐*α* expression,^35^ direct binding to NF‐κB and preventing subsequent proinflammatory gene expression, and increasing the expression of IκBβ, an NF‐κB inhibitor, to suppress NF‐κB transcription activity.^36^ Because NF‐κB activation is crucial in many cellular processes, tightly regulated NF‐κB signalling is required to fine‐tune the inflammatory responses. Counteracting an NF‐κB response is essential to prevent persistent NF‐κB activation, which may lead to chronic inflammation or tumour development. This is modulated by a number of negative regulators of NF‐κB, such as IKK1 and A20 (also known as TNFAIP3), A20‐binding inhibitor of NF‐κB, TNF receptor‐associated factor 1, cylindromatosis and microRNAs,^37^ some of which under the control of NF‐κB and thus act in a negative feedback loop. In addition, there is cross‐talk between NF‐κB signalling and the TGF‐β pathway that involve SMAD7 and TAK1.^38^ NF‐κB has a negative feedback to suppress TGF‐β signalling by activating inhibitory SMAD7.^39^ Our study shows that ELANE cells have a significant upregulation in *TNF*‐*α* and *TGF*‐*β1* expression and significant downregulation in *IL*‐*6*, *IL*‐*8* and *NF*‐*kB*‐*1*, compared with controls. Based on this evidence, we speculate that, in ELANE cells, the diminished *ELANE* and *SLPI* expression could lead to the upregulation of *TNF*‐*α* and *TGF*‐*β1*, whereas the downregulation of *NF*‐*kB1*, *IL*‐*6 and IL*‐*8* could be the result of extensive integration of NF‐κB signalling molecules, as well as cross‐talk between the NF‐κB and other signalling pathways. A schematic diagram of our proposed cellular pathomechanism is illustrated in Figure 5.

After LPS‐induced inflammatory responses, ELANE cells were larger compared with treated controls and untreated ELANE cells. Both the treated ELANE and control cells progressed faster to more advanced cell stages. LPS induces the expression of *TNF*‐*α*, *IL*‐*6*, *IL*‐*1B* and *IL*‐*8*.^40^ Correspondingly, treated ELANE and control cells showed significantly upregulated *SLPI*, *TNF*‐*α*, *NF*‐*kB1*, *IL*‐*6* and *IL*‐*8* expression compared with untreated cells, suggesting their response to inflammation. Comparing the fold changes between treated and untreated cells, treated ELANE cells exhibited a tremendous upregulation in *IL*‐*6* and *IL*‐*8* (24.07‐fold and 7.67‐fold increases respectively), compared with controls (1.57‐fold and 2.34‐fold). Moreover, the *TGF*‐*β1* expression in the LPS‐treated ELANE cells was significantly decreased (0.46‐fold change); however, that in controls was increased (1.67‐fold change). *NF*‐*kB1*, *IL*‐*6* and *IL*‐*8* are involved in cell proliferation, survival and inflammation, while *TGF*‐*ß1* is involved in growth arrest and anti‐inflammation. The differences between ELANE cells and controls in these inflammatory gene responses suggest that ELANE cells are highly responsive to inflammation.

Understanding cellular physiology is vital for studying human diseases, leading to a better understanding of pathophysiology and pathogenesis from cellular and molecular perspectives. Correlating cellular level changes to the phenotypic level elucidates disease pathomechanisms and leads to new approaches for early disease detection and treatment.

To conclude, the dental pulp cells with diminished *ELANE* and *SLPI* expression demonstrate reduced proliferation, migration, attachment, spreading, colony formation and wound healing, while having significantly elevated ROS, apoptosis and inflammatory responses. In addition to neutropenia that leads to oral infection, we show that severely downregulated neutrophil activity and *ELANE* and *SLPI* expression in dental cells could be involved in orodental infections. This study demonstrates that *ELANE* and *SLPI* play roles in proliferation, survival and inflammation in dental pulp cells.

## CONFLICT OF INTEREST

The authors declared no potential conflicts of interest with respect to the research, authorship and/or publication of this article.

## AUTHOR CONTRIBUTIONS

K. Sriwattanapong contributed to investigation, data analysis, drafted and critically revised the manuscript. N. Sa‐Ard‐Iam, L. Boonprakong, K. Subbalekha, V. Trachoo, N. Suratannon and V. Shotelersuk contributed to data interpretation and critically revised the manuscript. T. Porntaveetus was responsible for study conception, design, data acquisition and interpretation, drafted and critically revised the manuscript. All authors gave their final approval and agree to be accountable for all aspects of the work.

## Supporting information

Supplementary Material

## Data Availability

All data generated or analysed during this study are included in this published article (and its supplementary information files).
