# Supplementary material for: Reduced ELANE and SLPI expression compromises dental pulp cell activity
Source: Cell Prolif. 2021 Sep 27;54(11):e13132. doi: 10.1111/cpr.13132 (PMC8560611; doi:10.1111/cpr.13132)
Supplement: Supplementary file 1 — Supplementary Material [file CPR-54-e13132-s001.docx]

**Reduced ELANE and SLPI expression compromises dental pulp cell activity**

Kanokwan Sriwattanapong^1^, Noppadol Sa-Ard-Iam^2^, Lawan Boonprakong^3^, Keskanya Subbalekha^4^, Vorapat Trachoo^4^, Narissara Suratannon^5^, Thantrira Porntaveetus^1,*^, Vorasuk Shotelersuk^6,7^

^1^Genomics and Precision Dentistry Research Unit, Department of Physiology, Faculty of Dentistry, Chulalongkorn University, Bangkok, 10330, Thailand

^2^Center of Excellence in Periodontal Disease and Dental Implant, Immunology Research Center, Faculty of Dentistry, Chulalongkorn University, Bangkok, 10330, Thailand

^3^Oral Biology Research Center, Faculty of Dentistry, Chulalongkorn University, Bangkok 10330, Thailand

^4^Department of Oral and Maxillofacial Surgery, Faculty of Dentistry, Chulalongkorn University, Bangkok 10330, Thailand

^5^Pediatric Allergy & Clinical Immunology Research Unit, Division of Allergy and Immunology, Department of Pediatrics, Faculty of Medicine, Chulalongkorn University, King Chulalongkorn Memorial Hospital, The Thai Red Cross Society, Bangkok 10300, Thailand

^6^Center of Excellence for Medical Genomics, Medical Genomics Cluster, Department of Pediatrics, Faculty of Medicine, Chulalongkorn University, Bangkok, 10330, Thailand

^7^Excellence Center for Genomics and Precision Medicine, King Chulalongkorn Memorial Hospital, the Thai Red Cross Society, Bangkok, 10330, Thailand

**Appendix**

**
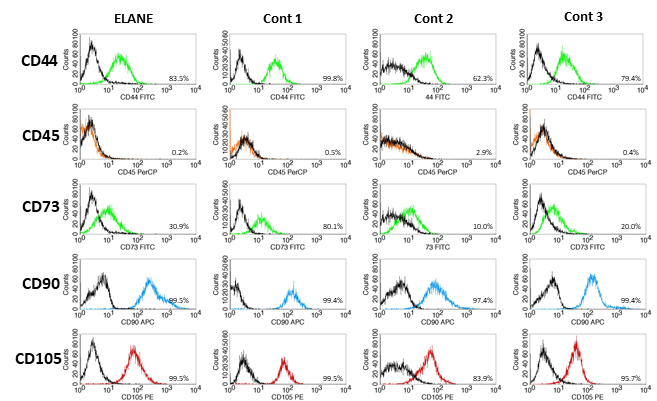
**

**Figure S1. Expression of mesenchymal stem cells markers.** ELANE and control cells were positive for CD44, CD90, CD105, and CD73; and negative for CD45.

**
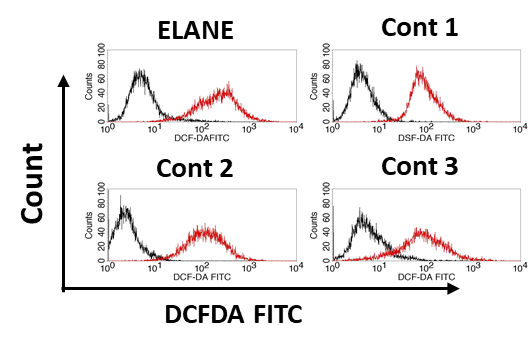
**

**Figure S2. Reactive oxygen species (ROS) level measured by flow cytometry using dichlorodihydrofluorescein diacetate** **(DCFDA).** ELANE cells demonstrated more ROS formation compared with controls.

**
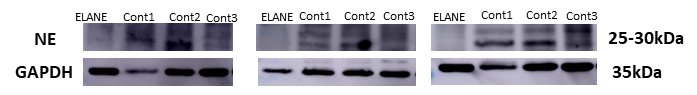
**

**
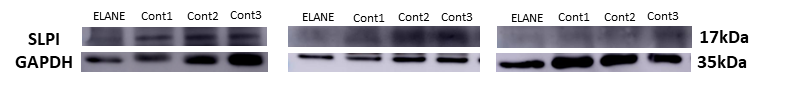
**

**Figure S3. Western blot images of ELANE and control cells.** The band intensities of NE and SLPI in ELANE cells were less than those in controls. Quantitative data of the protein bands was shown in Appendix Table 2.

**
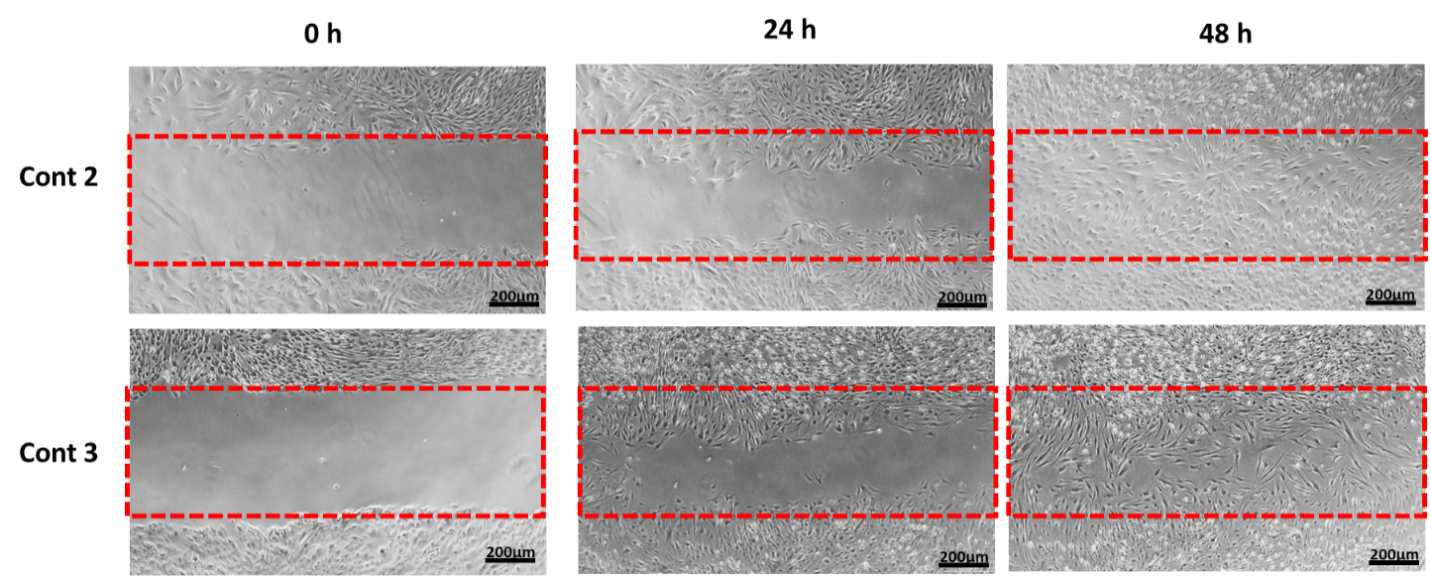
**

**Figure S4. Wound healing assay of controls 2 and 3.** The controls 2 and 3 demonstrated cell migration and gap closure.

**
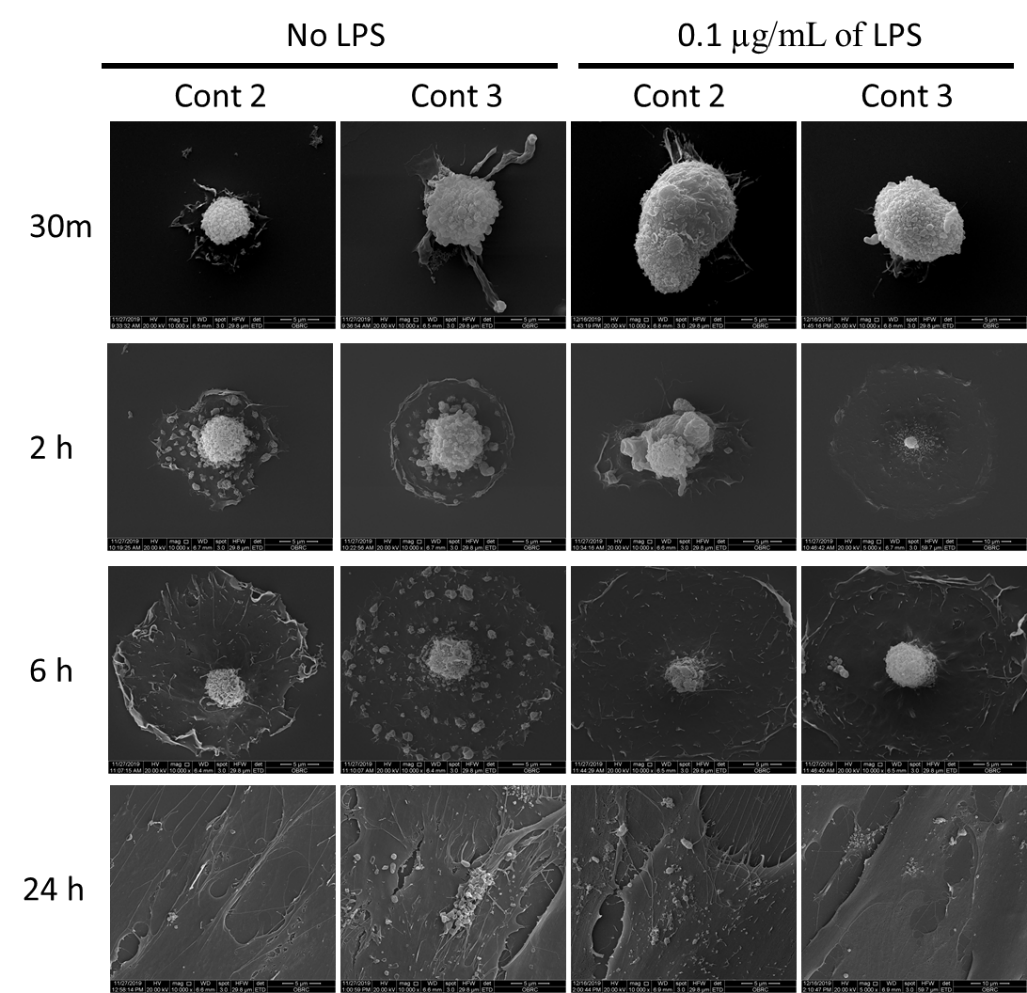
**

**Figure S5. Cell staging of controls 2 and 3 without LPS treatment.** Scanning electron microscopy showed that the control cells exhibited the filopodia and/or lamellopodia at 30 minutes. At 2 hours and 6 hours, the control cells formed extended circumferential lamellipodia. At 24 hours, the cells were flattened and completely spreading.

**
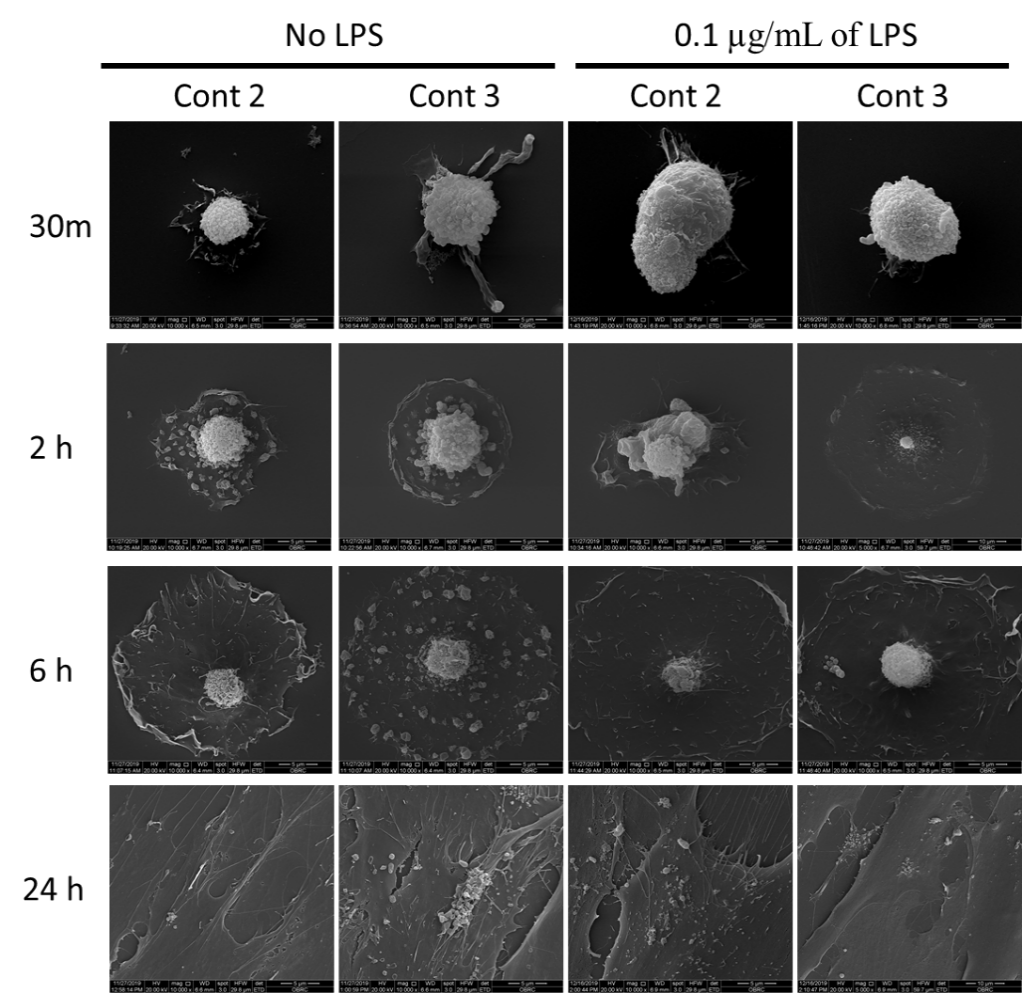

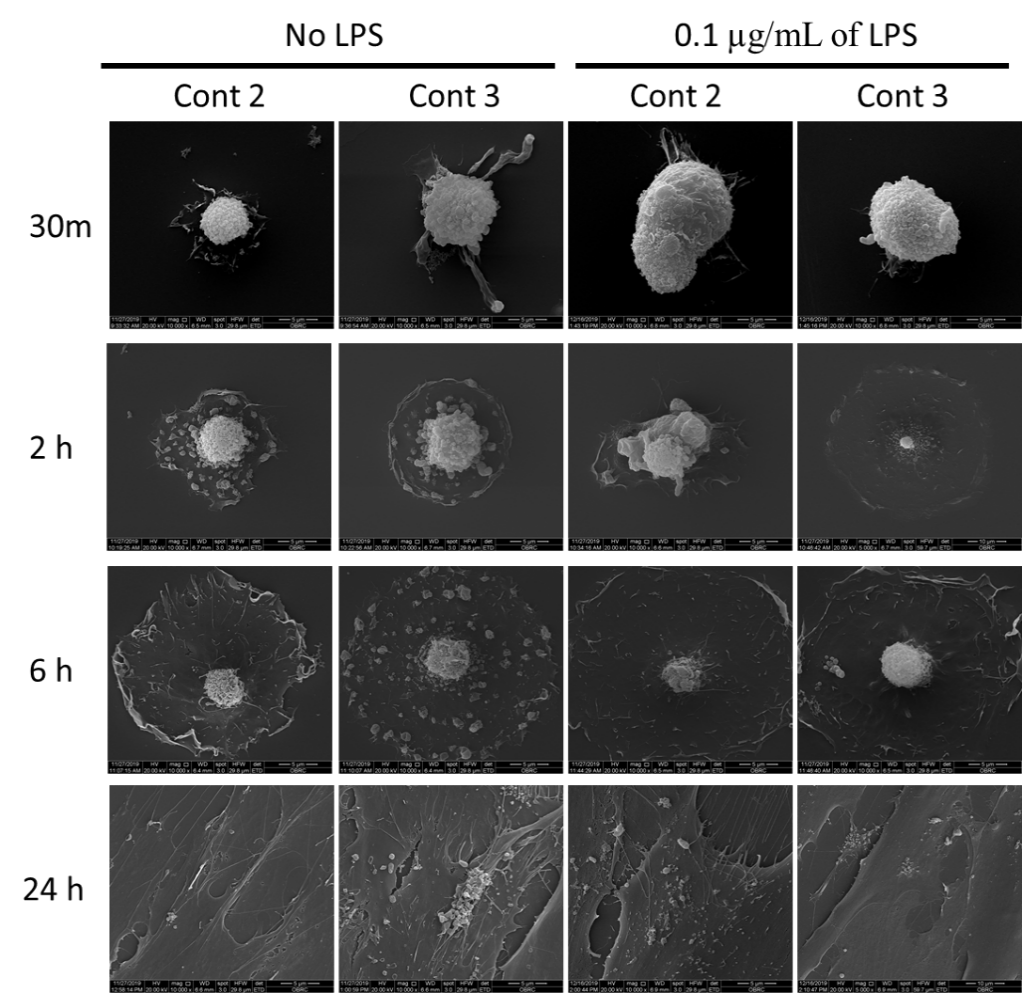
**

**Figure S6. Cell staging of controls 2 and 3 with LPS treatment.** Scanning electron microscopy demonstrated the ultrastructure and spreading of control cells at 30 minutes, 2 hours, 6 hours, and 24 hours.

**Table S1. Primer sequences.**

| **Primer Name** |  | **Primer Sequence (5’->3’)** |
| --- | --- | --- |
| ***ALP*** | Forward | CGA GAT ACA AGC ACT CCC ACT TC |
|  | Reverse | CTG TTC AGC TCG TAC TGC ATG TC |
| ***RUNX2*** | Forward | ATG ATG ACA CTG CCA CCT CTG A |
|  | Reverse | ATG GAG GGC GGA TTG GAA A |
| ***LPL*** | Forward | AGG ATG TGG CCC GGT TTA TC |
|  | Reverse | GCT CCA AGG CTG TAT CCC AA |
| ***PPAPγ*** | Forward | CCA GTG GTT GCA GAT TAC AAG TAT G |
|  | Reverse | TTG TAG AGC TGA GTC TTC TCA GAA TAA TAA G |
| ***ELANE*** | Forward | GCG TGG CGA A TG TAA ACG TC |
|  | Reverse | ACC CGT TGA GCT GGA GAA TC |
| ***SLPI*** | Forward | GTT GAC ACC CCA AAC CCA AC |
|  | Reverse | CAC AGG GGA AAC GCA GGA TT |
| ***MMP2*** | Forward | TTG ACG GTA AGG ACG GAC TC |
|  | Reverse | ACT TGC AGT ACT CCC CAT CG |
| ***Cyclin D1*** | Forward | ATC TCT GTA CTT TGC TTG CT |
|  | Reverse | AGT ACA TGG ATA TTC CCA AA |
| ***TGF-β1*** | Forward | GGA TAC CAA CTA TTG CTT CAG CT |
|  | Reverse | AGG CTC CAA ATG TAG GGG CAG GG |
| ***TNF-α*** | Forward | AGT GCT GGC AAC CAC TAA GAA |
|  | Reverse | AGA TGT CAG GGA TCA AAG CTG |
| ***IL-6*** | Forward | CCT GAA CCT TCC AAA GAT GGC |
|  | Reverse | CTG ACC AGA AGA AGG AAT GCC |
| ***IL-8*** | Forward | CCC AGT CTT GTC ATT GCC AG |
|  | Reverse | ACT GTG GAG TTT TGG CTG TTT T |
| ***NF-kβ1*** | Forward | GCC GTG GAG TAC GAC AAC ATC |
|  | Reverse | TTT GAG AAG AGC TGC CAG CC |
| ***β-actin*** | Forward | TGG AAC GGT GAA GGT GAC AG |
|  | Reverse | AAC AAC GCA TCT CAT ATT TGG AA |

**Table S2. Protein expression levels in ELANE and control cells.**

|  | **NE** | | |  |  |  | | **SLPI** | | | | |  | |  |
| --- | --- | --- | --- | --- | --- | --- | --- | --- | --- | --- | --- | --- | --- | --- | --- |
|  | No. 1 | No.2 | No.3 |  |  |  | | No. 1 | | No.2 | | No.3 |  | |  |
| ELANE | 8147.643 | 8553.338 | 8794.489 |  |  | ELANE | | 10994.342 | | 6248.572 | | 5666.167 |  | |  |
| Cont 1 | 12774.016 | 22703.258 | 20423.045 |  |  | Cont 1 | | 29545.007 | | 12603.551 | | 13085.492 |  | |  |
| Cont 2 | 17424.844 | 28103.329 | 24549.187 |  |  | Cont 2 | | 31904.53 | | 12257.392 | | 13142.016 |  | |  |
| Cont 3 | 9989.196 | 20765.317 | 27989.702 |  |  | Cont 3 | | 29065.602 | | 12208.401 | | 16726.593 |  | |  |
|  | | | | | | | | | | | | | | |  |
|  | **GAPDH** | | |  |  |  | | **GAPDH** | | | | |  | |  |
|  | No. 1 | No.2 | No.3 |  |  |  | | No. 1 | | No.2 | | No.3 |  | |  |
| ELANE | 27520.338 | 20967.459 | 21941.882 |  |  | ELANE | | 26518.137 | | 28701.572 | | 25794.35 |  | |  |
| Cont 1 | 11476.894 | 36650.066 | 21811.978 |  |  | Cont 1 | | 30115.794 | | 31131.631 | | 36065.238 |  | |  |
| Cont 2 | 21314.945 | 32994.279 | 29849.714 |  |  | Cont 2 | | 23982.853 | | 28303.744 | | 31914.409 |  | |  |
| Cont 3 | 17696.681 | 20561.413 | 24565.066 |  |  | Cont 3 | | 30355.581 | | 25170.43 | | 23727.317 |  | |  |
|  | | | | | | | | | | | | | | |  |
|  | **Ratio** | | |  |  |  | | **Ratio** | | | | |  | |  |
| ELANE | 0.296058973 | 0.407933932 | 0.400808326 |  |  | ELANE | | 0.41459707 | | 0.21770835 | | 0.219666981 |  | |  |
| Cont 1 | 1.1130203 | 0.619460221 | 0.936322465 |  |  | Cont 1 | | 0.98104692 | | 0.40484712 | | 0.362828383 |  | |  |
| Cont 2 | 0.817494204 | 0.851763695 | 0.822426205 |  |  | Cont 2 | | 1.33030586 | | 0.4330661 | | 0.411789421 |  | |  |
| Cont 3 | 0.564467202 | 1.009916828 | 1.13941082 |  |  | Cont 3 | | 0.95750439 | | 0.4850295 | | 0.70495088 |  | |  |
|  | | | | | | | | | | | | | | |  |
|  | **Relative density** | | | MWU |  |  | | **Relative density** | | | | | MWU | |  |
| ELANE | 1 | 1 | 1 |  |  | ELANE | | 1 | | 1 | | 1 |  | |  |
| Cont 1 | 3.75945471 | 1.518530753 | 2.336085364 | *P* < 0.05 |  | Cont 1 | | 2.36626591 | | 1.85958466 | | 1.651720168 | *P* < 0.05 | |  |
| Cont 2 | 2.761254612 | 2.087994226 | 2.051918962 | *P* < 0.05 |  | Cont 2 | | 3.20867162 | | 1.98920294 | | 1.874607728 | *P* < 0.05 | |  |
| Cont 3 | 1.906603931 | 2.475687233 | 2.842782311 | *P* < 0.05 |  | Cont 3 | | 2.30948178 | | 2.22788647 | | 3.209179986 | *P* < 0.05 | |  |
|  |  |  |  |  |  |  | |  | |  | |  |  | |  |
| No., number of experiement; MWU, Mann-Whitney U test. | | | | |  |  |  | |  | |  | | |  | |

**Table S3. Percentage of cell spreading and attachment in each stage at 2 and 6 hours in culture.**

| **Samples** | **Time** | **LPS (µg/mL)** | **Stage** | **Percentage** | **Standard error of mean** | **Time** | **LPS (µg/mL)** | **Stage** | **Percentage** | **Standard error of mean** |
| --- | --- | --- | --- | --- | --- | --- | --- | --- | --- | --- |
| **ELANE-SHED** | **2h** | 0 | 1 | 51.74 | 2.31 | **6h** | 0 | 1 | 23.62 | 2.10 |
|  |  |  | 2 | 28.88 | 1.57 |  |  | 2 | 53.27 | 2.07 |
|  |  |  | 3 | 14.99 | 0.69 |  |  | 3 | 16.08 | 0.52 |
|  |  |  | 4 | 4.39 | 0.29 |  |  | 4 | 7.04 | 0.49 |
|  |  | 0.1 | 1 | 51.03 | 1.89 |  | 0.1 | 1 | 22.15 | 0.63 |
|  |  |  | 2 | 35.70 | 1.65 |  |  | 2 | 39.34 | 0.82 |
|  |  |  | 3 | 12.32 | 0.65 |  |  | 3 | 22.15 | 0.78 |
|  |  |  | 4 | 0.95 | 0.25 |  |  | 4 | 16.36 | 0.63 |
| **Cont** |  | 0 | 1 | 3.63 | 0.26 |  | 0 | 1 | 3.68 | 0.41 |
|  |  |  | 2 | 9.15 | 0.99 |  |  | 2 | 2.71 | 1.12 |
|  |  |  | 3 | 49.41 | 3.30 |  |  | 3 | 52.37 | 1.68 |
|  |  |  | 4 | 37.76 | 2.87 |  |  | 4 | 41.23 | 1.22 |
|  |  | 0.1 | 1 | 6.98 | 1.48 |  | 0.1 | 1 | 0.00 | 0.00 |
|  |  |  | 2 | 13.86 | 1.34 |  |  | 2 | 5.34 | 0.81 |
|  |  |  | 3 | 34.52 | 1.62 |  |  | 3 | 48.89 | 5.87 |
|  |  |  | 4 | 44.58 | 1.39 |  |  | 4 | 45.40 | 6.35 |

**Table S4. Fold changes (ratio) of gene expression in the LPS-treated and untreated cells.**

| **Gene** | **ELANE** | **Control 1** | **Control 2** | **Control 3** | **Averaged Controls** |
| --- | --- | --- | --- | --- | --- |
| ***ELANE*** | **1.94** | 0.32 | 0.96 | 3.22 | **1.50** |
| ***SLPI*** | **3.53** | 0.19 | 2.20 | 9.63 | **4.00** |
| ***TGFB1*** | **0.46** | 1.80 | 1.25 | 1.96 | **1.67** |
| ***TNFa*** | **1.32** | 3.02 | 1.51 | 3.10 | **2.54** |
| ***NFkB1*** | **2.03** | 2.00 | 1.51 | 0.89 | **1.47** |
| ***IL-6*** | **24.07** | 0.91 | 2.42 | 1.37 | **1.57** |
| ***IL-8*** | **7.67** | 3.23 | 3.11 | 0.69 | **2.34** |
